# Effects of Guanxinning tablet on the gut microbiota and bile acid metabolism in mice with hyperlipidemia

**DOI:** 10.3389/fphar.2026.1754769

**Published:** 2026-03-27

**Authors:** Xincun Li, Jingya Ma, Yu Wang, Xiaoping Li, Chunsheng Zhu

**Affiliations:** Department of Chinese Medicine, The First Affiliated Hospital of Zhengzhou University, Zhengzhou, China

**Keywords:** bile acids, FXR, guanxinning tablet, gut microbiota, hyperlipidemia

## Abstract

**Introduction:**

Guanxinning tablet (GXNT), a traditional Chinese medicine preparation, has been found to improve lipid metabolism in patients with cardiovascular disease. However, the underlying mechanisms are still poorly understood. This study aims to determine whether the gut microbiota and bile acid (BA) metabolism is involved in the mechanisms by which GXNT ameliorates hyperlipidemia.

**Methods:**

The chemical composition of GXNT was characterized using UPLC-Q-TOF/MS. A mouse model of hyperlipidemia was established by feeding an high-fat diet (HFD), and GXNT or simvastatin was administered by gavage for 6 weeks. The impact of GXNT on hyperlipidemia was assessed by measuring markers related to lipid metabolism, liver injury and inflammation. Furthermore, 16S rDNA sequencing, targeted metabolomics, immunohistochemistry, molecular docking and western blot were used to investigate the underlying mechanisms.

**Results:**

GXNT treatment reduced blood lipid levels, improved liver injury, and mitigated hepatic inflammation in HFD-fed mice. GXNT also ameliorated the dysfunction of the intestinal barrier by upregulating the expression of zonula occludens-1 (ZO-1), occludin and claudin-1. Importantly, GXNT remodeled the gut microbiota in mice with hyperlipidemia, which was manifested by an increase in the abundance of Bacteroidota and Rikenellaceae*_RC9_gut_group*, as well as a decrease in the abundance of *Desulfovibrio*, *Monoglobus*, and *Streptococcus*. In addition, GXNT intervention altered the composition of fecal BAs and regulated BA metabolism by mediating the hepatic farnesoid X receptor (FXR)/small heterodimer partner (SHP) and intestinal FXR/fibroblast growth factor 15 (FGF15) axis.

**Conclusion:**

GXNT improved hyperlipidemia by altering the gut microbiota and regulating BA metabolism in HFD-fed mice. Our results provide a theoretical basis for the application of GXNT.

## 1 Introduction

The increasing prevalence of metabolic diseases has become an urgent global public health problem. Of these, hyperlipidemia is considered to be the main risk factor for atherosclerosis and cardiovascular disease (Cheng et al., 2024). Remarkably, with the changes of lifestyle, hyperlipidemia has caused high morbidity and mortality in many countries and regions. The latest data from the NHANES survey show that the prevalence of hyperlipidemia among young people aged 20 to 44 in the United States was 36.1% from 2017 to 2020 (Aggarwal et al., 2023). In addition, recent evidence suggests that the global number of deaths related to hyperlipidemia reached 4.3 million in 2019 (Chew et al., 2023). These findings indicate that hyperlipidemia poses a great threat to human health. Statins are generally considered the preferred drugs for preventing hyperlipidemia and reducing the risk of cardiovascular disease. Unfortunately, some patients are prone to statin intolerance, especially muscle symptoms (Stroes et al., 2015; Cholesterol Treatment Trialists, 2022). Consequently, it is necessary to develop novel alternative therapies for hyperlipidemia.

The gut microbiota, a group of microorganisms inhabiting the intestinal tract, has a profound impact on host metabolism and health. It has been reported that its structure and function are closely related to the development of various metabolic disorders, such as diabetes mellitus (Zhou et al., 2025), hyperuricemia (Zhu et al., 2023), non-alcoholic fatty liver disease (Jiang et al., 2024), and hyperlipidemia (Geng et al., 2024). Compared with individuals with low cholesterol, those with high cholesterol showed dysbiosis of the gut microbiota, characterized by an increased abundance of *Firmicutes* and a decreased abundance of *Bifidobacteria* (Kappel et al., 2023). Moreover, several specific bacterial taxa, including *Enterococcus*, Lachnospiraceae*_UCG-010*, and *unclassified_f_*Lachnospiraceae, have been confirmed to predict the outcomes of acute ischemic stroke patients with hyperlipidemia (Chen et al., 2022). Consistently, the aberrant composition of the bacterial community contributes to the development of hyperlipidemia in high-fat diet (HFD)-fed hamsters (Han et al., 2024). Of note, recent studies have found that *Blautia producta* is a potential probiotic for hyperlipidemia, which can significantly improve glycolipid metabolism in HFD-fed mice (Xu et al., 2023). Altogether, the above studies indicate that the gut microbiota is closely related to the pathological process of hyperlipidemia. Bile acids (BA), a group of end-products of cholesterol metabolism, play a pivotal role in lipid metabolism (Ying et al., 2018; Farr et al., 2020; Chen J. et al., 2024). For example, miR-378 promoted the synthesis of BA by regulating MAFG in the liver, thereby inhibiting the development of hypercholesterolemia (Sun et al., 2021). Consequently, the gut microbiota and BA metabolism are expected to become new targets for the treatment of hyperlipidemia.

The therapeutic effect of traditional Chinese medicine (TCM) in the management of hyperlipidemia has been extensively substantiated by a substantial body of clinical and pharmacological research (Tong et al., 2018; Tang et al., 2024; Han et al., 2025). Guanxinning tablet (GXNT) is a traditional Chinese medicine preparation produced by Zhengda Qingchunbao Pharmaceutical Co., Ltd., which is composed of *Salvia miltiorrhiza* Bunge (Danshen) and *Ligusticum chuanxiong* Hort. (Chuanxiong), are widely used in clinical practice to treat cardiovascular diseases. Accumulated evidence indicates that this medicine has significant effect in improving lipid metabolism (Li et al., 2023; Chen T. et al., 2024). However, the molecular mechanism underlying the anti-hyperlipidemic effects of GXNT remains unclear. In the present work, we established an HFD-induced hyperlipidemia mouse model and determined the therapeutic effects of GXNT on hyperlipidemia. In addition, 16S rDNA sequencing and targeted metabolomics analysis were performed to elucidate the potential molecular mechanisms underlying the anti-hyperlipidemic effects of GXNT.

## 2 Materials and methods

### 2.1 UPLC-Q-TOF/MS analysis of GXNT

GXNT was purchased from Zhengda Qingchunbao Pharmaceutical Co. Ltd. (Approval document number: Z20150028, Batch Number: 2406007), the composition of GXNT is shown in Table 1. The voucher specimens are stored in the Department of Traditional Chinese Medicine at the First Affiliated Hospital of Zhengzhou University. GXNT was crushed, ultrasonically extracted with 70% methanol and centrifuged. The supernatant was analyzed by UPLC-Q-TOF/MS. UPLC was performed on an Agilent 1290 system with an InfinityLab Poroshell 120 SB-Aq column (2.1 × 100 mm, 1.9 μm). The mobile phase comprised 0.1% formic acid (A) and acetonitrile (B) with a gradient: 2% B (0–3 min), 2%–25% B (3–18 min), 25%–40% B (18–30 min), 40%–95% B (30–38 min), and 95% B (38–40 min). Flow rate was 0.3 mL/min, column temperature was 40 °C, and injection volume was 2 μL. MS detection was carried out using an Agilent 6546 Q-TOF/MS with the following parameters: gas temperature, 350 °C; gas flow, 9 L/min; nebulizer, 35 psi; sheath gas temperature, 350 °C; sheath gas flow, 11 L/min; capillary voltage, 3000 V; nozzle voltage, 500 V; Skimmer1, 110 V, OctopoleRFPeak, 750. The MS scan range was 50–1500 m/z, and MS/MS scan range was 50–1500 m/z with collision energies of 10, 20, and 30 V.

**TABLE 1:** The composition of GXNT.

Chinese name	Family	Latin name	Plant part	Ratio in GXNT
Danshen	Lamiaceae	*Salvia miltiorrhiza* bunge	Root	1
Chuanxiong	Umbelliferae	*Ligusticum chuanxiong* hort	Root	1

### 2.2 Animals and experimental protocol

The animal design is shown in Figure 2A. Forty-eight SPF male ICR mice, with a weight of 20 ± 2 g, purchased from Beijing Sipeifu Biotechnology Co., Ltd. (NO. SCXK (Jing) 2019-0010). Adaptive feeding for 7 days and divided into 6 groups (n = 8): control (CON), model (MOD), simvastatin (ST), and high-, middle-, and low-dose GXNT (GXNT-H, GXNT-M, GXNT-L) groups. The CON group was given ordinary feed, while others were fed an HFD to induce hyperlipidemia. HFD (Catalog No. SFD018) consists of 58.6% basal diet, 15% lard, 20% sucrose, 5% casein, 1.2% cholesterol and 0.2% sodium cholate. The ST group were administered simvastatin (10 mg/kg/d, Suzhou Yushi Pharmaceutical Co., Ltd., Suzhou, China). The clinical adult dose of GXNT is 4560 mg/d. Using BSA conversion (factor≈9.1), the mice equivalent dose is 593 mg/kg/d. Therefore, GXNT groups were given 600, 300, and 150 mg/kg/d. CON and MOD groups received distilled water. Treatment lasted for 6 weeks. After fasting overnight, the mice were sacrificed by intraperitoneal injection of 1% sodium pentobarbital (100 mg/kg) with lidocaine in accordance with the AVMA Guidelines for the Euthanasia of Animals (2020 Edition). Blood was collected from the abdominal aorta followed by euthanasia via cervical dislocation. Cecal contents are used for 16S rDNA sequencing and BA metabolomics analysis. All experimental protocols were approved by the Ethics Committee of the First Affiliated Hospital of Zhengzhou University, with the approval No.2024-KY-0144.

### 2.3 Biochemical analysis

The activities of alanine aminotransferase (ALT) and aspartate aminotransferase (AST) were detected by fully automatic biochemical analyzer. The levels of total cholesterol (TC) (E-BC-K109-M), triglycerides (TG) (E-BC-K251-M), high-density lipoprotein cholesterol (HDL-C) (E-BC-K221-M), low-density lipoprotein cholesterol (LDL-C) (E-BC-K205-M) and D-Lactic Acid (D-LA) (E-BC-K002-M) in the serum were measured using colorimetric assays using commercial kits (Elabscience, Wuhan, China). The concentrations of diamine oxidase (DAO) (E-EL-M0412) and lipopolysaccharides (LPS) (CSB-E13066m) in the serum were detected by ELISA kits (Elabscience, Wuhan, China). All procedures followed the manufacturers’ protocols.

### 2.4 Histological analysis of the liver

The mouse liver was taken and photographed on the coordinate paper to document morphology and color. Part of the liver was fixed in polyformaldehyde, dehydrated, paraffin-embedded, and then hematoxylin-eosin (H&E) staining was performed (HR0721, Henan Dinghan Biotechnology Co., Ltd, Henan, China). Another portion was frozen in liquid nitrogen, OCT-embedded, and cryosectioning (8 μm-thick) for oil red O staining to assess lipid deposition. ImageJ software is used to analyze the image and calculate the average grayscale value.

### 2.5 Measurement of hepatic proinflammatory factors

The proinflammatory factors interleukin 1β (IL-1β) (E-EL-M0037), interleukin 6 (IL-6) (E-EL-M0044) and tumor necrosis factor alpha (TNF-α) (E-EL-M3063) in the liver were measured using ELISA kits (Elabscience, Wuhan, China). Prior to detection, liver tissue was washed with pre-cooled PBS (0.01 M, pH 7.4) to remove residual blood. The liver tissue was weighed and fully homogenized in PBS on ice, and then centrifugation at 2 °C–8 °C for 5–10 min at 5000×g. The supernatant was then collected for further analysis.

### 2.6 Immunohistochemistry

Mouse colon tissue was fixed, dehydrated, made transparent, and paraffin-embedded, then cut into 4 μm thick. The sections were dewaxed, hydrated, antigen recovery, and endogenous peroxidase blocking by using an Immunohistochemical staining kit (HR0725, Henan Dinghan Biotechnology Co., Ltd, Henan, China). Subsequently, they were incubated with primary antibodies (Occludin, 1:100, 13409-1-AP; Claudin-1, 1:100, 13050-1-AP; zonula occludens-1 (ZO-1), 1:1000, 21773-1-AP, all purchased from Proteintech, USA) overnight at 4 °C, then treated with enhancer and enzyme-labeled secondary antibody. Concurrently, 3,3′-diaminobenzidine was used to visually stain and hematoxylin was followed by counterstaining, ethanol dehydration, and neutral resin mounting. The ImageJ software was used for analysis under the light microscope.

### 2.7 16S rDNA sequencing

Fecal samples (0.2–0.5 g) were added to centrifuge tubes containing the extract lysate for grinding as a pretreatment step. Nucleic acids were extracted from the pretreated samples with OMEGA Soil DNA Kit (M5635-02, Omega Bio-Tek, Norcross, GA, USA). The V3-V4 region-specific primers (338F: 5′-barcode + ACT​CCT​ACG​GGA​GGC​AGC​A-3′ and 806R: 5′-GGACTACHVGGGTWTCTAAT-3′) of bacterial 16S rRNA gene were used for PCR amplification. The PCR products were quantified using the Quant-iT PicoGreen dsDNA Assay Kit (P7589, Invitrogen, Carlsbad, CA, USA) and mixed as needed. The library was constructed using the TruSeq Nano DNA LT Library Prep Kit (Illumina, USA) and subjected to quality inspection and quantification. For the qualified library, the NovaSeq 6000 SP Reagent Kit (500 cycles) was used for 2 × 250 bp double-end sequencing on the Illumina NovaSeq machine. QIIME2 and R software were utilized for quality control and analysis of the sequencing data. Simpson and Faith_pd were used to assess the ɑ diversity, principal coordinates analysis (PCoA) was used to assess the β diversity, and linear discriminant analysis effect size (LEfSe) analysis was used to screen the biomarkers of bacterial taxa for each group. LDA score >4 and *p* < 0.05 were used to identify the biomarkers.

### 2.8 Targeted metabolomics analysis

EXion LC Liquid chromatography (AB SCIEX, USA) and AB6500 Plus (AB SCIEX, USA) were used for metabolomics analysis. The chromatographic column was ACQUITY UPLC® BEH C18 (2.1 × 100 mm, 1.7 μm, Waters, USA). Injection volume was 5 μL, the column temperature was 40 °C, and the mobile phase was A-0.01% formic acid water, B-acetonitrile. The gradient elution conditions were 0–4 min, 25% B; 4–9 min, 25%–30% B; 9–14 min, 30%–36% B; 14–18 min, 36%–38% B; 18–24 min, 38%–50% B; 24–32 min, 50%–75% B; 32–33 min, 75%–90% B; 33–35.5 min, 90%-25% B. The flow rate was 0.25 mL/min. Mass Spectrometry Conditions were as follows: electrospray ionization (ESI) source, negative ionization mode. The ion source temperature was 500 °C, the ion source voltage was −4500 V, the collision gas was 6 psi, the curtain gas 30 psi, and the atomizing gas and auxiliary gas were both 50 psi. Scans performed using multiple reaction monitoring. Principal component analysis (PCA) and orthogonal partial least squares discriminant analysis (OPLS-DA) were used to evaluate the overall distribution trend among groups, PLS-DA overfitting test was used to assess the reliability, and Spearman’s correlation analysis was used to assess the relationship between gut microbiota and metabolites. Variable importance in the projection (VIP) value >1 and *p* < 0.05 were used to identify differential metabolites.

### 2.9 Western blot

The liver and colon were washed with PBS, dissolved in RIPA (including PMSF) (R0010, Solarbio, Beijing, China) for 30 min, and then the supernatant collected by centrifuged at 12000 g for 20 min. Each group took 30 μg of total protein for electrophoresis and transfer to membrane, and then blocked with 5% skim milk powder for 1 h. Finally, incubate with anti-I overnight at 4 °C and then incubate with anti-II for 1 h. The anti-I information are as follows: anti-ZO-1 antibody (1:5000, 21773-1-AP, Proteintech, USA), anti-Occludin antibody (1:2000, 13409-1-AP, Proteintech, USA), anti-Claudin-1 antibody (1:1000, 13050-1-AP, Proteintech, USA), anti-fibroblast growth factor 15 (FGF15) antibody (1:1000, ab225942, abcam, UK), anti-farnesoid X receptor (FXR) antibody (1:1000, 25055-1-AP, Proteintech, USA), anti-cholesterol 7α-hydroxylase (CYP7A1) antibody (1:1000, DF2612, Affnity, Jiangsu, China), anti-cholesterol 12α-hydroxylase (CYP8B1) antibody (1:1000, DF4762, Affinity, Jiangsu, China), anti-small heterodimer partner (SHP) antibody (1:5000, 82503-1-RR, Proteintech, USA), and anti-GAPDH antibody (1:5000, 10494-1-AP, Proteintech, USA). The anti-II was HRP-conjugated Goat Anti-Rabbit IgG (H + L) (1:8000, SA00001-2, Proteintech, USA). The Chemi Doc XRS + imaging System (Bio-Rad, Hercules, CA, USA) was used for imaging after exposure to the ECL chemiluminescence reagent (P0018S, Beyotime, Shanghai, China). The ImageJ software was used for bands analysis.

### 2.10 Molecular docking

The 3D crystal structure of FXR was obtained from the RCSB Protein Data Bank (https://www.rcsb.org). The 3D structure of the ligand GXNT was retrieved from the PubChem database (https://pubchem.ncbi.nlm.nih.gov). Protein preparation was performed using AutoDock Tools1.5.6 by removing water molecules and co-crystallized ligands, followed by the addition of polar hydrogen atoms, assignment of partial charges, and merging of non-polar hydrogens. The ligand was also prepared using AutoDock Tools. Molecular docking was conducted using AutoDock Tools 1.5.6, and binding free energy was calculated to evaluate the binding affinity between FXR and GXNT chemical composition. Docking poses and protein-ligand interactions were visualized and analyzed using PyMOL.

### 2.11 Statistical analysis

Graphpad Prism 9.0 (GraphPad Software, LLC, USA) was used to process the data. Measurement data were expressed as mean ± standard deviation. One-way analysis of variance was used for comparison among multiple groups when the data followed the normal distribution and met the homogeneity of variance. *P < 0.05* was considered statistically significant.

## 3 Results

### 3.1 Chemical composition of GXNT by UPLC-Q-TOF/MS analysis

**FIGURE 1:**
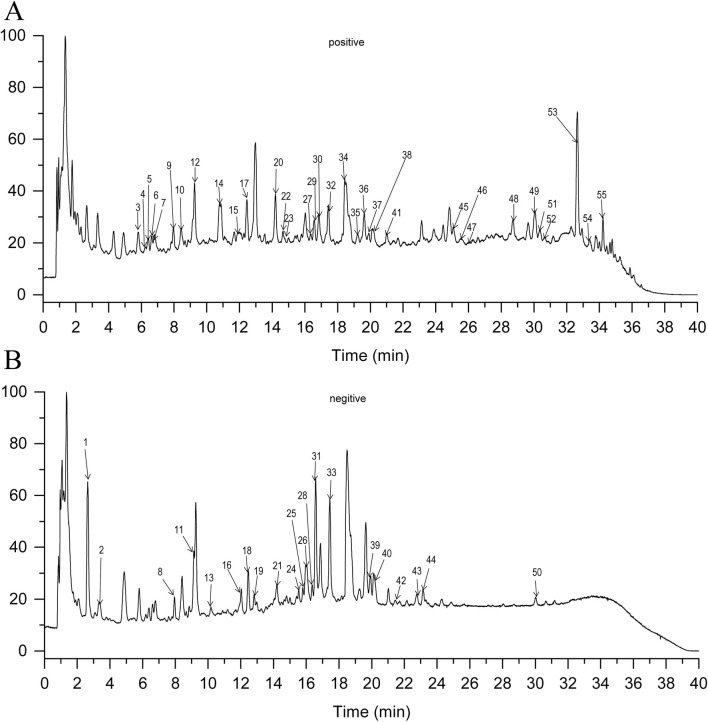
Chemical composition of GXNT by UPLC-Q-TOF/MS analysis. The total ion chromatogram in positive **(A)** and negative ion modes **(B)**.

The chemical composition of GXNT were initially identified by UPLC-Q-TOF/MS analysis, and a total of 55 compositions were identified. Detailed information of these compositions is presented in Table 2, mainly including cryptotanshinone, dihydrotanshinone, senkyunolide A, senkyunolide K, salvianolic acid A, salvianolic acid B, chlorogenic acid, caffeic acid and other compositions. In addition, the corresponding ion chromatogram of GXNT is shown in Figure 1.

**TABLE 2:** Chemical composition of GXNT by UPLC-Q-TOF/MS.

No.	Metabolites	Formula	m/z	Selective ion	RT (min)
1	Danshensu	C_9_H_10_O_5_	197.0455	[M-H]^−1^	2.644
2	Protocatechuic acid 7-O-glucoside	C_13_H_16_O_9_	315.0724	[M-H]^−1^	3.412
3	Neochlorogenic acid	C_16_H_18_O_9_	355.1029	[M + H]^+1^	5.799
4	Danshensu-9-O-[apiosyl-(1→6)]-glucoside	C_20_H_28_O_14_	491.1402	[M + H]^+1^	6.424
5	Isomer of danshensu-9-O-[apiosyl-(1→6)]-glucoside	C_20_H_28_O_14_	491.1402	[M + H]^+1^	6.641
6	Isomer of danshensu-9-O-[apiosyl-(1→6)]-glucoside	C_20_H_28_O_14_	491.1402	[M + H]^+1^	6.724
7	Isomer of danshensu-9-O-[apiosyl-(1→6)]-glucoside	C_20_H_28_O_14_	491.1402	[M + H]^+1^	6.825
8	Icariside F2	C_18_H_26_O_10_	401.1451	[M-H]^−1^	7.999
9	Chlorogenic acid glucoside	C_22_H_28_O_14_	517.1548	[M + H]^+1^	8.001
10	Caffeic acid	C_9_H_8_O_4_	181.0498	[M + H]^+1^	8.435
11	Cryptochlorogenic acid	C_16_H_18_O_9_	353.0876	[M-H]^−1^	9.259
12	Chlorogenic acid	C_16_H_18_O_9_	355.1029	[M + H]^+1^	9.277
13	Senkyunolide R	C_12_H_16_O_5_	239.0920	[M-H]^−1^	10.16
14	Senkyunolide J	C_12_H_18_O_4_	227.1283	[M + H]^+1^	10.778
15	Senkyunolide N	C_12_H_18_O_4_	227.1283	[M + H]^+1^	11.696
16	Isochlorogenic acid A	C_25_H_24_O_12_	515.1193	[M-H]^−1^	12.061
17	Ferulic acid	C_10_H_10_O_4_	195.0639	[M + H]^+1^	12.430
18	Chuanxiongoside A	C_23_H_36_O_12_	503.2128	[M-H]^−1^	12.527
19	Salvianolic acid F	C_17_H_14_O_6_	313.0716	[M-H]^−1^	12.837
20	Senkynolide H	C_12_H_16_O_4_	207.1017	[M + H]^+1^	12.980
21	Methyl-4-hydroxy-3,5-dimethoxycinnamate	C_12_H_14_O_5_	237.0771	[M-H]^−1^	14.188
22	Senkynolide I	C_12_H_16_O_4_	207.1017	[M + H]^+1^	14.231
23	Senkyunolide D	C_12_H_14_O_4_	223.0969	[M + H]^+1^	14.635
24	Isomer of isochlorogenic acid A	C_25_H_24_O_12_	515.1193	[M-H]^−1^	15.581
25	Salvianolic acid D	C_20_H_18_O_10_	417.0828	[M-H]^−1^	15.802
26	Lithospermic acid	C_27_H_22_O_12_	537.1038	[M-H]^−1^	15.990
27	Chuanxiongoside B	C_23_H_36_O_12_	505.2264	[M + H]^+1^	16.058
28	Dedihydro-salvianolic acid B	C_36_H_28_O_16_	715.1302	[M-H]^−1^	16.357
29	Isomer of isochlorogenic acid A	C_12_H_16_O_2_	517.1348	[M + H]^+1^	16.358
30	Sagerinic acid	C_36_H_32_O_16_	741.1433	[M + Na]^+1^	16.575
31	Rosmarinic acid	C_18_H_16_O_8_	359.0778	[M-H]^−1^	16.607
32	Salvianolic acid L	C_36_H_30_O_16_	736.1868	[M + H]^+1^	16.867
33	Salvianolic acid A	C_26_H_22_O_10_	493.1143	[M-H]^−1^	17.450
34	Salvianolic acid B	C_36_H_30_O_16_	736.1868	[M + H]^+1^	18.444
35	Salvianolic acid Y	C_36_H_30_O_16_	736.1874	[M + H]^+1^	19.319
36	Salvianolic acid K	C_36_H_30_O_16_	736.1874	[M + H]^+1^	19.620
37	Salvianolic acid E	C_36_H_30_O_16_	736.1874	[M + H]^+1^	19.736
38	Monomethyl lithospermate	C_28_H_24_O_12_	553.1341	[M + H]^+1^	19.870
39	3‴-deoxy-salvianolic acid B	C_36_H_30_O_15_	701.1497	[M-H]^−1^	19.944
40	9-methyl lithospermate	C_37_H_32_O_16_	731.1609	[M-H]^−1^	20.127
41	Z-ligustilide	C_12_H_14_O_2_	191.1066	[M + H]^+1^	20.204
42	Senkyunolide F	C_12_H_14_O_3_	205.0873	[M-H]^−1^	21.445
43	Senkyunolide K	C_12_H_14_O_3_	205.0872	[M-H]^−1^	22.696
44	9,12,13-Trihydroxy-10-octadecenioc acid	C_18_H_34_O_5_	329.2333	[M-H]^−1^	23.171
45	Senkyunolide A	C_12_H_16_O_2_	193.1223	[M + H]^+1^	24.708
46	Senkyunolide M	C_16_H_22_O_4_	279.1586	[M + H]^+1^	25.075
47	3-Butylphthalide	C_12_H_14_O_2_	191.1066	[M + H]^+1^	25.63
48	Hydroxytanshinone IIA	C_20_H_20_O_5_	341.1392	[M + H]^+1^	28.45
49	Dihydrotanshinone	C_18_H_14_O_3_	279.1011	[M + H]^+1^	29.662
50	Cryptotanshinone	C_19_H_22_O_4_	313.1442	[M-H]^−1^	30.061
51	Chuanxiongnolide L5	C_24_H_30_O_6_	415.2119	[M + H]^+1^	30.096
52	Danshixinkun B	C_18_H_16_O_3_	281.1162	[M + H]^+1^	30.43
53	Cryptotanshinone	C_19_H_20_O_3_	297.1491	[M + H]^+1^	32.632
54	Tanshinone I	C_18_H_12_O_3_	277.0861	[M + H]^+1^	32.957
55	Tanshinone IIA	C_19_H_18_O_3_	295.1336	[M + H]^+1^	34.241

### 3.2 Effects of GXNT on lipid metabolism

**FIGURE 2:**
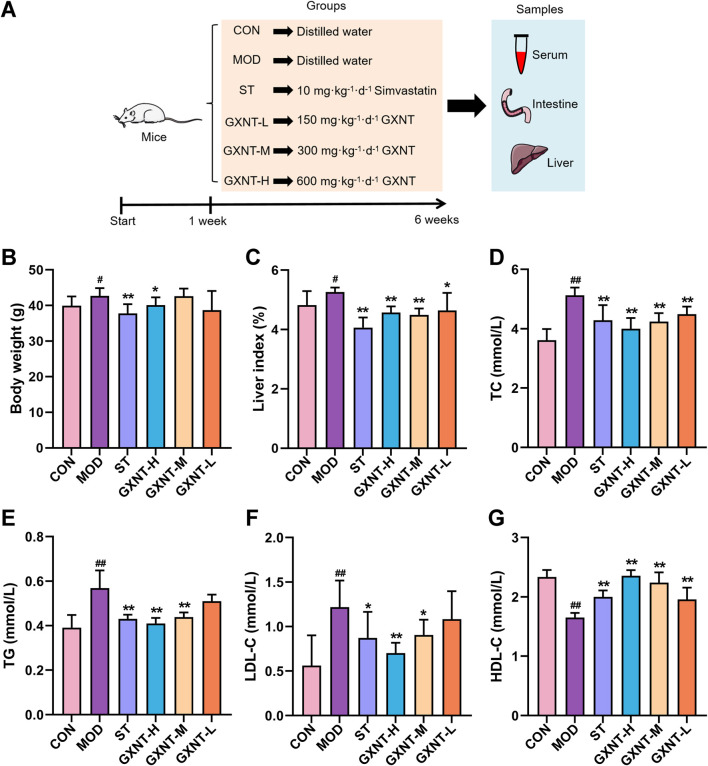
Effects of GXNT on body weight and lipid metabolism of HFD-fed mice. **(A)** The animal experimental design; **(B)** Body weight in the sixth week; **(C)** Liver index; **(D–G)** Serum TC, TG, LDL-C, and HDL-C levels. ^#^ *P* < 0.05, ^##^ *P* < 0.01 compared with the CON group; *P < 0.05, **P < 0.01 compared with the MOD group.

As shown in Figures 2B,C, the body weight and liver index of the mice in the MOD group increased significantly after 6 weeks of HFD feeding (*p* < 0.05, Figures 2B,C). Administration of high-dose GXNT markedly reduced the body weight (*p* < 0.05), whereas high, medium, and low doses of GXNT significantly reduced the liver index (*p* < 0.05, *p* < 0.01). Subsequently, serum lipid levels were detected, and the results indicated that GXNT had similar lipid-lowering effect as simvastatin. Specifically, compared with the CON group, the serum TC, TG, and LDL-C of mice in the MOD group increased and HDL-C decreased (*p* < 0.01, Figures 2D–G). GXNT treatment could reduce TC, TG and LDL-C in HFD-fed mice (*p* < 0.05, *p* < 0.01), and increase HDL-C in a dose-dependent manner (*p* < 0.01). In summary, these findings indicate that GXNT can ameliorate lipid metabolism in mice with hyperlipidemia.

### 3.3 GXNT alleviated liver injury and inflammation

**FIGURE 3:**
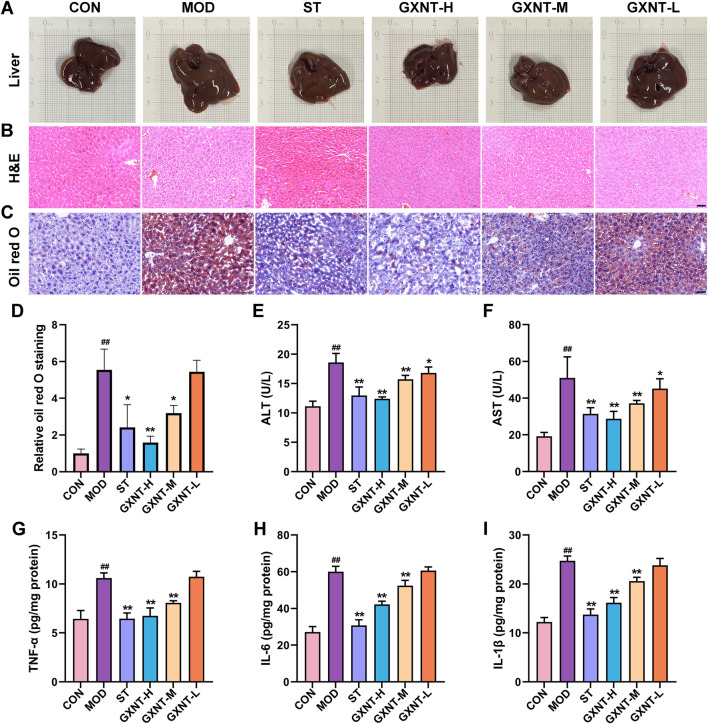
Effects of GXNT on liver injury and inflammation in HFD-fed mice. **(A)** Images of livers; **(B,C)** H&E and oil red O staining of liver samples (×200 magnification, scale bar: 100 μm); **(D)** Relative Oil red O grayscale values; **(E,F)** The activities of serum ALT and AST; **(G–I)** The levels of TNF-α, IL-6, and IL-1β in the liver. ^##^ *P* < 0.01 compared with the CON group; *P < 0.05, **P < 0.01 compared with the MOD group.

Compared to the CON group, the liver color of the MOD group appeared paler (Figure 3A). Nevertheless, the GXNT-H group exhibited a more reddish and healthy liver. As shown in Figures 3B,E,H staining revealed the presence of vacuoles in the cytoplasm, nuclear fragmentation, and inflammatory cell infiltration in the liver form the MOD mice. In parallel, oil red O staining found obvious accumulation of lipid droplets (Figures 3C,D). Of note, GXNT ameliorated these histological alterations to a certain extent. Moreover, liver function-related indicators were measured. As shown in Figures 3E,F, ALT and AST activities from the MOD group were increased compared to the CON group, indicating that the liver function of HFD-fed mice was impaired. Administration of GXNT significantly reduced the ALT and AST activities of mice in the MOD group. By assaying TNF-α, IL-6, and IL-1β, we aimed to investigate whether an HFD induced inflammation in the liver. As shown in Figures 3G–I, TNF-α, IL-6 and IL-1β from the MOD were significantly higher than those of the CON (*p* < 0.01), which could be reversed by high and medium doses of GXNT. Overall, GXNT treatment improved inflammatory responses and liver injury in HFD-fed mice.

### 3.4 GXNT ameliorated the intestinal barrier

**FIGURE 4:**
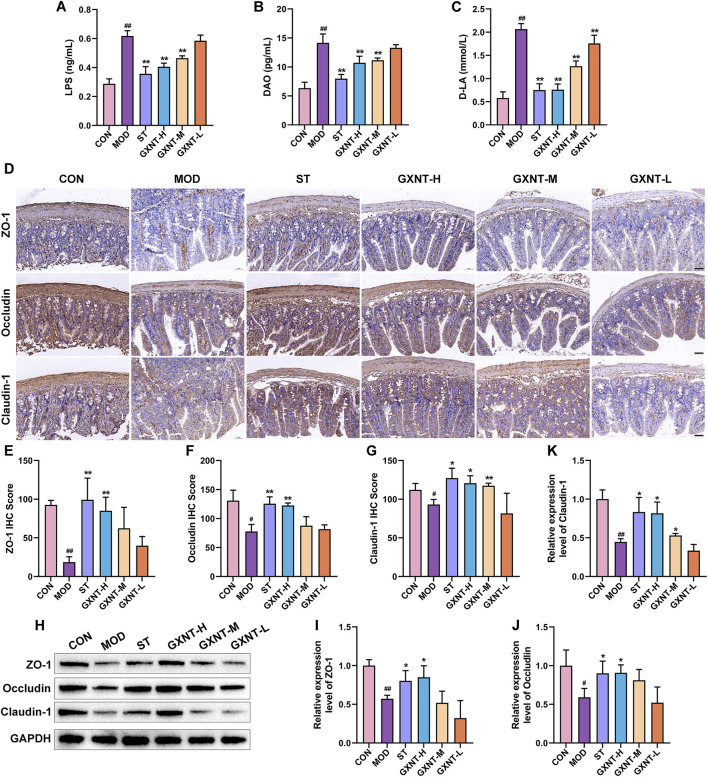
GXNT ameliorated the dysfunction of the intestinal barrier. **(A–C)** The levels of serum LPS, DAO and D-LA; **(D)** Immunohistochemical staining of ZO-1, occludin and claudin-1 in intestinal tissues; **(E–G)** Fluorescence intensity analysis of ZO-1, occludin and claudin-1; **(H–K)** The protein levels of ZO-1, occludin and claudin-1 in intestinal tissues. ^#^ *P* < 0.05, ^##^ *P* < 0.01 compared with the CON group; *P < 0.05, **P < 0.01 compared with the MOD group.

Detection of serum LPS, DAO and D-LA levels to evaluate intestinal barrier function. As shown in Figures 4A–C, compared with the CON group, LPS, DAO and D-LA were significantly increased in the MOD group (*p* < 0.01), implying that the disruption of intestinal barrier integrity and increased permeability are associated with the development of hyperlipidemia. Interestingly, their levels were reduced after GXNT intervention. To further investigate the role of GXNT on the intestinal, tight junction (TJ) proteins in intestinal tissues, including ZO-1, occludin and claudin-1 were used to evaluate the intestinal barrier. Immunohistochemistry results discovered that the MOD group showed lower levels of ZO-1, occludin, and claudin-1 compared to the CON group (*p* < 0.05, *p* < 0.01, Figures 4D–G). On the contrary, high and medium doses of GXNT caused a significant increase in the levels of these TJ proteins (*p* < 0.05, *p* < 0.01). Western blot analysis further confirmed that GXNT reversed the downregulation of ZO-1, occludin and claudin-1 induced by HFD feeding (*p* < 0.05, Figures 4H–K), suggesting that this medicine may alleviate hyperlipidemia by restoring intestinal barrier function.

### 3.5 GXNT remodeled the gut microbiota

**FIGURE 5:**
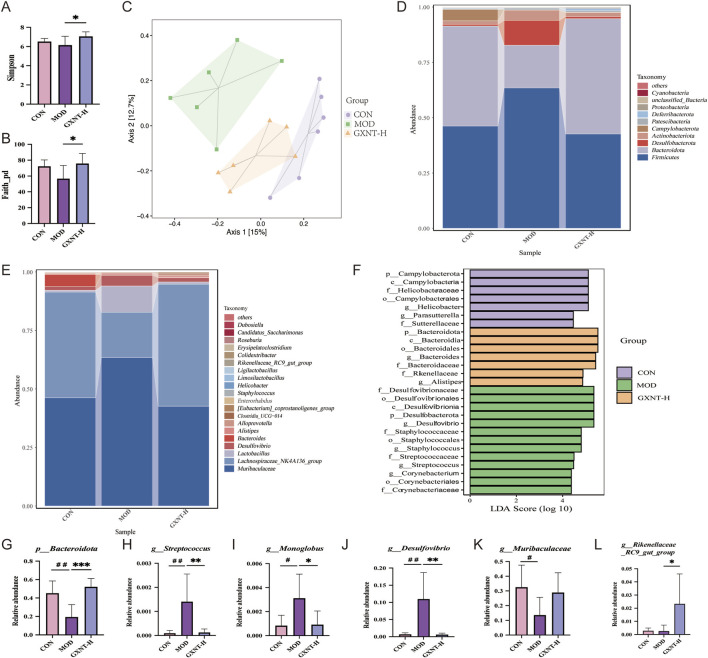
GXNT reshaped the gut microbiota in HFD-fed mice. **(A,B)** The indexes of Simpson and Faith_pd; **(C)** PCoA of the gut microbiota among all groups; **(D,E)** Relative abundance of the gut microbiota at the phylum and genus levels; **(F–K)** Relative abundance of six key bacteria at the genus level. **(L)** LDA scores derived from LEfSe analysis. ^#^ *P* < 0.05, ^##^ *P* < 0.01 compared with the CON group; *P < 0.05, **P < 0.01, ***P < 0.001 compared with the MOD group.

To investigate the impact of GXNT on the gut microbiota in hyperlipidemia, the composition of microbiota was analyzed using 16S rDNA sequencing. Given the significant therapeutic effect of high-dose GXNT in hyperlipidemic mice, the GXNT-H group was chosen for further investigation. As depicted in Figures 5A,B, the indexes of Simpson and Faith_pd were significantly elevated in the GXNT-H, indicating that GXNT-H increased the ɑ diversity. Meanwhile, β diversity was determined by principal coordinate analysis (PCoA). The results demonstrated a notable distance in the bacterial composition of the three groups of samples from CON, MOD, and GXNT-H (Figure 5C), implying that the gut microbiota was dysregulated in hyperlipidemic, and that its composition was significantly regulated by GXNT-H intervention. Subsequently, phylum and genus levels were used to determine the relative abundance of each group. Notably, Firmicutes and Bacteroidota are the predominant phylum, and *Muribaculaceae* and *Lactobacillus* are the predominant genus among the three groups (Figures 5D,E). Compared with the CON group, the relative abundance of Bacteroidota and *Muribaculaceae* in the MOD group was lower (Figures 5F,K), while the relative abundance of *Streptococcus*, *Monoglobus*, and *Desulfovibrio* was increased (*p* < 0.05, *p* < 0.01, Figures 5G–I). Interestingly, GXNT-H significantly reversed the levels of four of these bacterial taxa, including Bacteroidota*, Desulfovibrio*, *Monoglobus*, and *Streptococcus* (*p* < 0.05, *p* < 0.01). Additionally, GXNT-H significantly increased the abundance of Rikenellaceae*_RC9_gut_group* in hyperlipidemic mice (*p* < 0.05, Figure 5J). The biomarkers of bacterial taxa for each group were identified using linear discriminant analysis effect size (LEfSe) analysis. Our results found that *o_Bacteroidales*, *p_Bacteroidota* and *c_Bacteroidia* were enriched in the GXNT-H group, *f_*Desulfovibrionaceae, *o_Desulfovibrionales* and *c_Desulfovibrionia* were enriched in the MOD group, and *p_Campylobacterota*, *c_Campylobacteria* and *f_*Helicobacteraceae were enriched in the CON group (Figure 5L). Collectively, these data suggest that GXNT can ameliorate the dysbiosis of the gut microbiota in mice with hyperlipidemia.

### 3.6 GXNT regulated the BA composition in the feces

**FIGURE 6:**
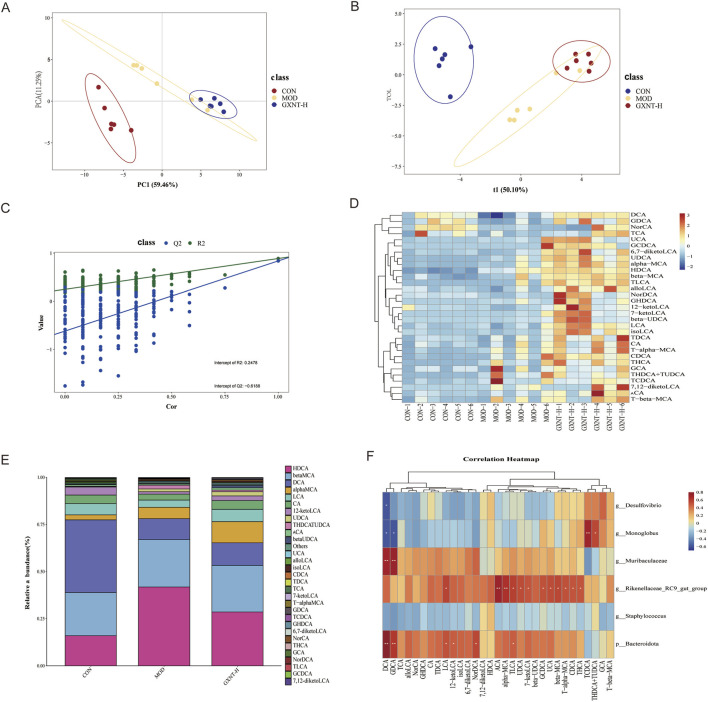
GXNT regulated the BA composition in the feces of HFD-fed mice. **(A,B)** PCA and OPLS-DA of BAs among all groups; **(C)** PLS-DA overfitting test; **(D)** Heatmap displaying the most significant differences in BAs; **(E)** Relative abundance of BAs that significantly altered between the groups; **(F)** Spearman’s correlation analysis of differential microbiota and BAs.

BAs play a crucial role in hyperlipidemia. Therefore, we performed targeted metabolomics analysis to assess the BA profile in the feces from the CON, MOD, and GXNT-H. PCA and OPLS-DA showed obvious separation between CON, MOD, and GXNT-H (Figures 6A,B). Moreover, Figure 6C showed that the model was reliable. As depicted in Figure 6D, the levels of 31 BAs showed significant variations among the CON, MOD, and GXNT-H groups. Further studies showed that the MOD group had higher abundance of hyodeoxycholic acid (HDCA) and lower abundance of lithocholic acid (LCA) and cholic acid (CA) than the CON group (Figure 6E). After GXNT intervention, the abundance of HDCA was decreased, whereas LCA and CA was increased. Spearman’s correlation analysis found that *Muribaculaceae* and Bacteroidota were positively correlated with glycodeoxycholic acid (GDCA) and deoxycholic acid (DCA) levels, and Rikenellaceae*_RC9_gut_group* was positively correlated with allocholic acid (ACA) and alpha-muricholic acid (alpha-MCA) levels (Figure 6F). *Streptococcus* was positively correlated with taurochenodeoxycholic acid (TCDCA) levels and was negatively correlated with DCA levels. Meanwhile, *Desulfovibrio* displayed a negative association with DCA levels, and *Monoglobus* showed a negative association with DCA and GDCA levels. Overall, GXNT could regulate the BA composition in HFD-fed mice.

### 3.7 Molecular docking of GXNT chemical components and FXR

**FIGURE 7:**
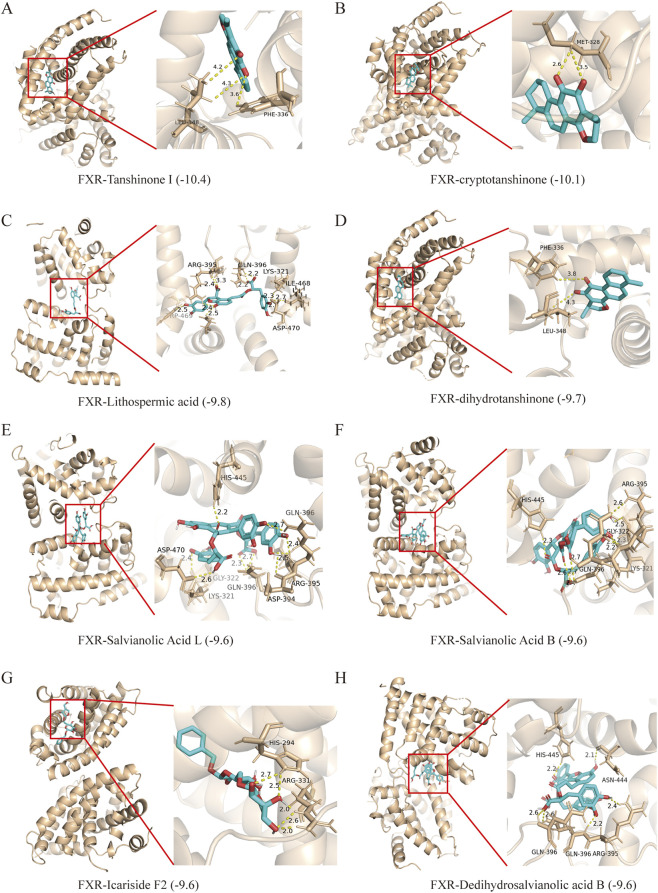
Molecular docking diagram of GXNT chemical composition and FXR. Visual results of molecular docking in tanshinone I **(A)**, cryptotanshinone **(B)**, lithospermic acid **(C)**, dihydrotanshinone **(D)**, salvianolic acid L **(E)**, salvianolic acid B **(F)**, icariside F2 **(G)** and dedihydro-salvianolic acid B **(H)** with FXR.

Molecular docking results showed that the binding energy of 43 active components were <5.5 kcal/mol (see Supplementary Material 2), among which tanshinone I (Figure 7A), cryptotanshinone (Figure 7B), lithospermic acid (Figure 7C), dihydrotanshinone (Figure 7D), salvianolic acid L (Figure 7E), salvianolic acid B (Figure 7F), icariside F2 (Figure 7G) and dedihydro-salvianolic acid B (Figure 7H) had high binding activity with FXR (≤9.6 kcal/mol). The docking demonstration diagram is shown in Figure 7.

### 3.8 Effects of GXNT on BA metabolism

**FIGURE 8:**
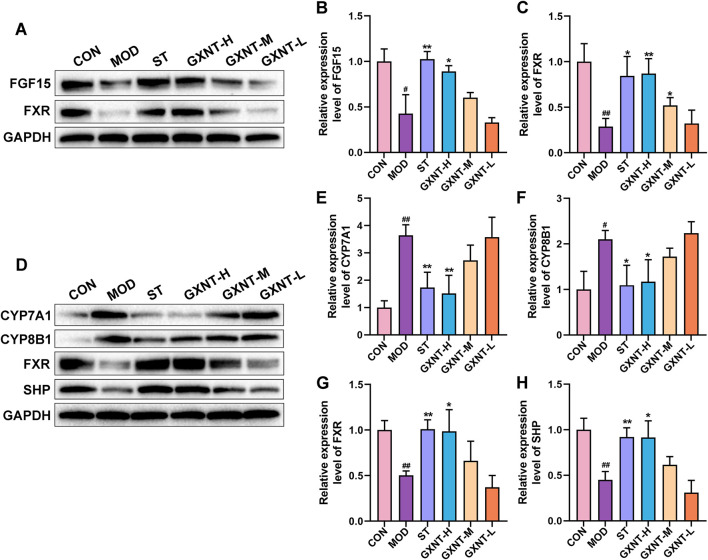
Effects of GXNT on BA metabolism. **(A–C)** The protein levels of FGF15 and FXR in the ileum; **(D–H)** The protein levels of CYP7A1, CYP8B1, FXR and SHP in the liver. ^#^ *P* < 0.05, ^##^ *P* < 0.01 compared with the CON group; *P < 0.05, **P < 0.01 compared with the MOD group.

To further determine the effects of GXNT on BAs, we examined the expression of BA metabolism-related proteins in the ileum and liver. As shown in Figures 8A–C, the expression of FGF15 and FXR was significantly downregulated in the ileum of the MOD group compared to the CON group (*p* < 0.05, *p* < 0.01). Conversely, high dose of GXNT upregulated the expression of FGF15 (*p* < 0.05), and high and medium dose of GXNT upregulated the expression of FXR (*p* < 0.05, *p* < 0.01). Furthermore, compared to normal mice, HFD-fed mice exhibited increased expression of CYP7A1 and CYP8B1 (*p* < 0.05, *p* < 0.01, Figures 8D–F), as well as decreased expression of FXR and SHP in the liver (*p* < 0.01, Figures 8G,H). After GXNT intervention, the expression trends of these proteins were significantly reversed. In summary, our results indicate that GXNT can regulate the BA metabolism in HFD-fed mice.

## 4 Discussion

Hyperlipidemia is considered as a major risk factor for cardiovascular disease, which poses a huge threat to human health. Therefore, it is imperative to develop new agents to prevent and treat hyperlipidemia. In recent years, TCM and its active components have attracted people’s attention due to their remarkable effect and low toxicity. Of note, GXNT, a formulation consisting of *S. miltiorrhiza* and *L. chuanxiong*, has been demonstrated to effectively improve lipid metabolism (Yang et al., 2022). It has been reported that *S. miltiorrhiza* can reduce TC, TG and LDL, increase HDL, and alleviate weight gain in HFD-fed rats (Ai et al., 2022). Tanshinone IIA, a bioactive metabolite from *S. miltiorrhiza*, ameliorated hepatic lipid deposition by regulating miR-33a and SREBP-2 (Jia et al., 2016). Similarly, tetramethylpyrazine, a metabolite derived from *L. chuanxiong*, could improve glucose and lipid metabolism in diabetic rats by regulating the PI3K/Akt/GLUT-4 pathway (Rai et al., 2019). Moreover, phthalides from *L. chuanxiong* could protect mice against diabetic nephropathy (Qi et al., 2024). In this study, UPLC/MS combined with molecular docking found that tanshinone I, cryptotanshinone, lithospermic acid, dihydrotanshinone, salvianolic acid L and other ingredients may be the active ingredients for GXN to exert its anti-hyperlipidemic effect. Overall, the above research results suggest that *S. miltiorrhiza* and *L. chuanxiong* may be promising therapeutic agents for metabolic diseases. However, the underlying mechanism of action of GXNT on hyperlipidemia remains unknown.

In the present study, a mouse model of hyperlipidemia was established by feeding an HFD. Hyperlipidemic mice showed significant increases in body weight and liver index, as well as elevated serum TC, TG, and LDL-C concentrations and reduced HDL-C levels, verifying successful establishment of the hyperlipidemia model. After GXNT intervention, the trend of changes in these indicators was reversed. H&E staining showed that vacuoles appeared in the liver cytoplasm of mice in the MOD, nucleus fragmentation, and inflammatory cell infiltration; oil red O staining showed that lipid droplets accumulated in the MOD. The activies of ALT and AST are important indicators that reflect liver function. Compared to the CON, the activities of ALT and AST were elevated in the MOD. These data indicate that hyperlipidemic mice exhibit obvious liver injury. Interestingly, GXNT not only alleviated histological changes in the liver but also reduced the activities of ALT and AST. In addition, hyperlipidemia is closely associated with inflammatory responses in the liver (Martínez-Clemente et al., 2010; Yang et al., 2025; Zhang X. Y. et al., 2025). Recent studies have found that the levels of IL-1β, TNF-α, and caspase-1 are elevated in the liver of mice with tyloxapol-induced hyperlipidemia, while scopoletin can reduce the levels of these inflammatory factors (Zhang J. et al., 2025). Analogously, GXNT could reduce the levels of TNF-α, IL-6, and IL-1β, indicating that GXNT alleviate hepatic inflammation triggered by hyperlipidemia.

Growing evidence suggests that intestinal homeostasis plays a crucial role in various disease physiological processes (Chen et al., 2025). The intestinal barrier is mainly composed of mucus, epithelial cells, and tight junction (TJ) proteins, which maintain intestinal homeostasis by preventing harmful substances from entering the systemic circulation. Of these, TJ proteins, including ZO-1, occludin and claudin-1, are widely recognized as key markers of barrier integrity. Interestingly, dysfunction of the intestinal barrier is involved in the development of various metabolic diseases, such as diabetes (Peng et al., 2025), hyperuricemia (Zhang T. et al., 2025), and hyperlipidemia (Pan et al., 2025). For example, obese mice exhibited increased body weight, elevated blood glucose and lipid levels, as well as hepatic steatosis, which was associated with disruption of the intestinal barrier (Kang et al., 2023). Previous studies have found that xylooligosaccharides can improve the function of the intestinal barrier in HFD-fed mice by upregulating the expression of TJ proteins (Li et al., 2021). In our study, the levels of LPS, DAO and D-LA in the MOD were higher than the CON group, and the levels of ZO-1, occludin, and claudin-1 were lower than those in the CON group, implying that hyperlipidemia may contribute to the dysfunction of the intestinal barrier. However, GXNT effectively reduced intestinal permeability by restoring the integrity of the intestinal barrier.

The gut microbiota is a complex and dynamic ecosystem within the intestinal tract, whose diversity and abundance significantly influence host metabolism (Rondanelli et al., 2025). Strikingly, gut microbiota dysbiosis is closely associated with the pathogenesis of hyperlipidemia (Tao et al., 2024). Yan et al. found that *Bacteroides* species were responsible for the sulfonation of cholesterol (Yan et al., 2024). Moreover, urolithin A, a gut microbiota-derived metabolite, improved cholesterol metabolism by inhibiting the transcription of SREBP1 and SREBP2 (Xu et al., 2024). The above findings indicate that the gut microbiota serves as a critical regulator in the development of hyperlipidemia. To further clarify the mechanism of GXNT, we analyzed the fecal microbiota profile in mice with hyperlipidemia. Our results showed that GXNT treatment not only increased the diversity of the gut microbiota, but also modulates its abundance in hyperlipidemic mice. Specifically, compared to the CON group, the relative abundance of Bacteroidota was reduced in the MOD group. GXNT intervention significantly reversed its relative abundance. Bacteroidota is one of the most abundant bacterial species in the human gut. In HFD-fed mice, the abundance of Bacteroidota was significantly reduced (Li et al., 2022). Importantly, supplementation with galactooligosaccharides and sodium alginate significantly attenuated body weight gain and improved lipid metabolism, which was closely associated with an increase in the levels of Bacteroidota, and *Lactobacillus* (Li et al., 2022). Another study found that fermented yogurt effectively decreased body weight and hepatic lipid droplet formation in obese mice by increasing the abundance of *Blautia*, *norank_f_Muribaculaceae* and Rikenellaceae*_RC9_gut_group* (Sun et al., 2024). Consistent with these findings, our results demonstrated that GXNT could increase the abundance of certain potentially beneficial taxa, such as Bacteroidota and Rikenellaceae*_RC9_gut_group*, which might be the potential molecular mechanisms by which GXNT improved hyperlipidemia.

*Streptococcus* is generally considered a conditional pathogen, and studies have found that the abundance of *Streptococcus* in the intestine of rats with type 2 diabetes is increased (Bi et al., 2025). Analogously, mice with hyperlipidemia showed higher levels of *Streptococcus* and *Faecalibaculum*, as well as reduced levels of *Akkermansia* and *Roseburia* (Deng et al., 2023). Polysaccharides from Gougunao tea could improve inflammation and lipid metabolism in hyperlipidemic mice by reversing the levels of these microbiota (Deng et al., 2023). In addition, dendrobine improved hyperlipidemia by regulating the composition of the gut microbiota, as indicated by a decrease in the levels of *Desulfovibrio*, and an increase in the abundance of *Actinobacteria* (Song et al., 2024). Consistently, our data showed that GXNT significantly reduced the relative abundance of *Streptococcus* and *Desulfovibrio*, indicating that this traditional Chinese metabolite preparation can inhibit the proliferation of harmful microbiota in mice with hyperlipidemia. *Monoglobus* has been reported to be beneficial for host energy metabolism (Wang Y. et al., 2024). However, our study revealed a reduction in its abundance following GXNT intervention. Thus, more research is necessary to elucidate the role of *Monoglobus* in hyperlipidemia.

It is widely recognized that BAs, as the end-products of cholesterol metabolism in hepatocytes, play a crucial role in the solubilization and digestion of lipid-soluble nutrients from food. Upon entering the intestines, primary BAs are further metabolized by the gut microbiota into a wide array of secondary BAs. These BAs serve as signaling molecules and are involved in multiple host physiological processes, especially energy metabolism. In our study, GXNT regulated the BA composition in HFD-fed mice, manifesting as an increase in the abundance of LCA and CA, as well as a decrease in the abundance of HDCA. We further examined the expression of BA metabolism-related proteins in the ileum and liver. On one hand, GXNT treatment increased the expression of FXR and FGF15 in the intestines of hyperlipidemic mice. On the other hand, this medicine reduced the expression of CYP7A1 and CYP8B1, and increased the expression of FXR and SHP in the liver. These findings suggest that GXNT may ameliorate hyperlipidemia by modulating BA metabolism. The hepatic FXR/SHP axis and intestinal FXR/FGF15 axis have been demonstrated to be implicated in BA homeostasis (Thomas et al., 2008). FXR enhanced the expression of SHP by activating the SHP promoters, thereby inhibiting BA biosynthesis through the downregulation of CYP7A1 and CYP8B1 expression (Goodwin et al., 2000). FGF15 serves as a critical link between the liver and the intestine (Inagaki et al., 2005). Specifically, FXR could promote the expression of FGF15 in the intestine, which subsequently inhibited the expression of CYP7A1 in the liver by activating FGF receptor 4 (FGFR4) (Inagaki et al., 2005). Our results showed that GXNT activated both FXR/SHP axis and FXR/FGF15 axis, and inhibited the expression of CYP7A1 and CYP8B1, indicating that GXNT suppressed BA biosynthesis in mice with hyperlipidemia. Interestingly, activation of the FXR/SHP pathway inhibited miR-802 expression, leading to improved glucose tolerance and reduced hepatic TG levels in obese mice (Seok et al., 2021). Another study found that FXR not only reduced fatty acid synthesis by regulating the expression of lipogenic genes in the liver but also decreased lipid absorption by reducing BA levels (Clifford et al., 2021). Obeticholic acid, a FXR agonist, has been demonstrated to reduce serum HDL-C levels and hepatic cholesterol levels in hamsters with hyperlipidemia (Dong et al., 2017). In summary, these findings suggest that BA metabolism-related proteins, such as FXR, SHP, FGF15, and CYP7A1, may serve as potential targets for hyperlipidemia treatment.

Secondary BA glycoursodeoxycholic acid (GUDCA) is a natural FXR antagonist. Sun et al. found that GUDCA could improve insulin resistance and body weight gain by inhibiting FXR signaling in the gut of obese mice (Sun et al., 2018). Analogously, gut microbiota-derived isodeoxycholic acid (isoDCA) and 7-oxo-deoxycholic acid (7-oxo-DCA) participated in the development of colorectal cancer by acting as regulators of FXR (Dong et al., 2024). Furthermore, recent studies indicated that sodium butyrate improved cholestatic liver injury by increasing the expression of hepatic FXR (Wang T. et al., 2024). These findings reveal that microbial metabolites may serve as mediators between the gut microbiota and FXR signaling regulation. Therefore, further research is still needed to explore the potential molecular mechanisms by which GXNT regulates the FXR signaling.

## 5 Conclusion

**FIGURE 9:**
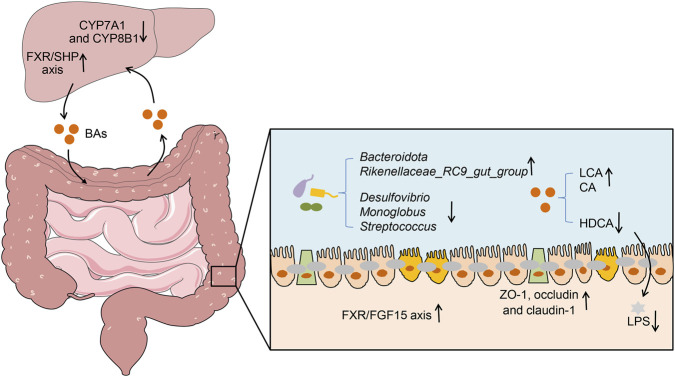
Potential mechanisms of GXNT in improving hyperlipidemia.

Our study revealed that GXNT effectively lowered blood lipid levels, improved liver injury, and mitigated hepatic inflammation in mice with hyperlipidemia. Furthermore, GXNT ameliorated the function of intestinal barrier by upregulating the expression of TJ proteins. Regarding the potential mechanisms, GXNT not only alleviated gut microbiota dysbiosis but also altered the composition of BAs in the feces of HFD-fed mice. At the same time, GXNT regulated BA metabolism by regulating the hepatic FXR/SHP axis and intestinal FXR/FGF15 axis (Figure 9). These findings provide a theoretical basis for the application of GXNT in the treatment of hyperlipidemia, particularly in statin-intolerant patients. Nevertheless, there are certain limitations in our study. In the future, the molecular mechanisms by which GXNT ameliorates hyperlipidemia will be further elucidated through fecal microbiota transplantation and FXR knockout models. We used only samples from the high-dose GXNT group for microbiome and metabolomics analysis, making it difficult to elucidate the relationship between the microbiota and BA changes and dose. Therefore, future studies will include multiple dose groups in microbiome/metabolomics analyses. Furthermore, additional clinical trials are required to assess the impact of GXNT on lipid metabolism.

## Data Availability

The data presented in the study are deposited in the MetaboLights Human Metabolome Database, accession number MTBLS14095. This data can be found here: https://www.ebi.ac.uk/metabolights/MTBLS14095.
