# Supplementary material for: Effects of Guanxinning tablet on the gut microbiota and bile acid metabolism in mice with hyperlipidemia
Source: Front Pharmacol. 2026 Mar 27;17:1754769. doi: 10.3389/fphar.2026.1754769 (PMC13066125; doi:10.3389/fphar.2026.1754769)
Supplement: Supplementary file 2 [file Supplementaryfile1.doc]

EXion LC Liquid chromatography (AB SCIEX, USA) and AB6500 Plus (AB SCIEX, USA) were used for metabolomics analysis. The chromatographic column was ACQUITY UPLC® BEH C18 (2.1×100 mm, 1.7 μm, Waters, USA). Injection volume was 5 μL, the column temperature was 40 °C, and the mobile phase was A-0.01% formic acid water, B-acetonitrile. The gradient elution conditions were 0-4 min, 25% B; 4-9 min, 25-30% B; 9-14 min, 30-36% B; 14-18 min, 36-38% B; 18-24 min, 38-50% B; 24-32 min, 50-75% B; 32-33 min, 75-90% B; 33-35.5 min, 90-25% B. The flow rate was 0.25 mL/min. Mass Spectrometry Conditions were as follows: electrospray ionization (ESI) source, negative ionization mode. The ion source temperature was 500 °C, the ion source voltage was -4500 V, the collision gas was 6 psi, the curtain gas 30 psi, and the atomizing gas and auxiliary gas were both 50 psi. Scans performed using multiple reaction monitoring.
